# Evaluation of the use of blood in surgeries as a tool to change patterns for requesting blood product reserves

**DOI:** 10.6061/clinics/2019/e652

**Published:** 2019-04-16

**Authors:** Sibia Soraya Marcondes, Antônio Roberto Carrareto, Maria da Penha Zago-Gomes, Maria do Perpétuo Socorro Vendramini Orletti, Anisia Carla Zucoloto Loureiro Novaes

**Affiliations:** I Hospital Universitario Cassiano Antonio Moraes, Vitoria, ES, BR; II Faculdade de Medicina, Escola Superior de Ciencias da Santa Casa de Misericordia de Vitoria, Vitoria, ES, BR; III Centro de Ciências da Saude, Universidade Federal do Espirito Santo, Vitoria, ES, BR; IV Centro de Hematologia e Hemoterapia do Espirito Santo (HEMOES), Vitoria, ES, BR

**Keywords:** Blood Transfusion, Red Blood Cell Transfusion, Elective Surgical Procedures, Blood Banks, Blood Grouping, Cross-Matching

## Abstract

**OBJECTIVES:**

Thirty to sixty percent of prepared blood products are not transfused. Blood reserves for surgeries lead to many unused blood products, which increases hospital costs. The aim of this study is to identify the request and use profiles of blood products for elective surgeries in different surgical specialties, the influence of surgery time and demographic, clinical, and laboratory variables on the number of red blood cells (RBCs) used and to calculate the rate of transfused patients (RTP) and cross-matched and transfused (C/T) RBCs.

**METHODS:**

Observational and prospective studies. Sociodemographic, clinical and quantitative data on the request and use of blood products were collected. The influence of the data on the use of RBCs was examined by binary logistic regression. Chi-square, one-way ANOVA and *Kruskal-Wallis* tests were utilized to compare the data among the specialties.

**RESULTS:**

In total, 822 procedures were included. Most of the requested blood products were not used, even 24 hours postoperatively. Of the 2,483 RBC units, 314 were transfused, leaving 87.6% unused; however, cardiac, digestive tract, vascular, gynecologic, urologic and thoracic surgery procedures transfused 50%, 25%, 16.5%, 11%, 9.5% and 8.1% of requested RBCs, respectively. The factors that influenced the transfusions were age, time of surgery and cardiac surgeries. The RTP was >10% in 22 surgical types and <1% in 24 surgical types, and 88% of samples presented a C/T ratio >2.5.

**CONCLUSION:**

The RTP and C/T ratios can guide RBC requests in the preoperative period. Knowing the standard of use of blood products and developing protocols enables the optimization of reserves, reduction of costs and improvement of care.

## INTRODUCTION

Inventory management and rational blood use have become essential for efficacy in hemotherapy procedures 1,2. Data from developed countries show that only 40-70% of units of blood prepared for transfusion are actually transfused 3. The blood reserves for surgical patients contribute to this statistic, which requires expenditures with inputs and human resources and prevents the use of these reserved units for other hospital demands, leading to the loss of blood products due to expiration 4. Chawla, Kakepoto and Khan 5 demonstrated a potential savings of 62.88% of blood products with better management of blood reserves for procedures. Additionally, better management of blood reserves implemented in Michigan 6 reduced blood product disposal from 6.5% to 4.5%. Therefore, the management and optimization of blood reserves for surgeries are essential for cost reduction and healthcare improvements 8.

One of the parameters used to manage blood reserves is the rate of transfused patients (RTP), which uses calculations and estimates to assess the need for transfusion in surgeries 6,9-11. For surgical procedures with RTP values greater than 10%, a previous blood product compatibility evaluation is recommended. For values between 1 and 10%, blood typing is recommended. In turn, for RTP values less than 1%, no previous preparation is recommended 9,11. Another widely used index is the ratio between cross-matched and transfused (C/T) RBC units, in which values greater than 2 indicate an excessive number of requested compatible units 9,10,12,13.

Stanworth et al. 14 demonstrated that the use of blood products in elective surgeries was the third most common cause for blood product indications (14.7%), behind only cases that included victims of trauma (20.6%) and patients with malignancies (17.3%). Through C/T ratio evaluations, Collins et al. 4 demonstrated that most surgery services requested too much blood for the preoperative reserve for elective procedures. The evaluation of the use of red blood cell (RBC) concentrates in health services and the consumption among different medical specialties has provided data that can guide the application of “good practice” strategies in hemotherapy 14.

However, the University Hospital of Espírito Santo did not perform an evaluation of their blood consumption profile, and this analysis is essential for management improvement in hemotherapy. Therefore, the present study aimed to identify the profile of the request and use of blood products for elective surgeries in the different surgical specialties of a university hospital to identify the influence of demographic, clinical and laboratory variables and surgical time on the number of RBCs used in elective surgeries and to calculate the RTP and C/T ratios.

## MATERIALS AND METHODS

This study was an observational, prospective study that evaluated elective surgeries in which blood reserves were requested at the Cassiano Antônio Moraes University Hospital, Federal University of Espírito Santo (Universidade Federal do Espírito Santo – Brazil), from 02/01/2015 to 01/31/2016. This study was approved by the ethics committee on hospital research (CAAE 39820414.8.0000.5071).

Patients older than 15 years of age who underwent elective surgeries in different specialties that had preoperative reserved blood products and presented laboratory results of hemogram or prothrombin time (PT) and activated partial thromboplastin time (APTT) were included. Patients were excluded if they underwent emergency surgeries; were under the age of 15 years, since pediatric patients do not characterize the hospital population; or underwent an elective surgery without laboratory data collection because the absence of laboratory data would impair the analysis of the risk variables.

Data were collected from six surgical specialty case types and included sociodemographic data; laboratory data, including hemoglobin (Hb), hematocrit (Ht), platelet count, PT, APTT and irregular antibody screening (IAS) levels; blood products data, including the requested and used number of RBC units; and duration of surgery in minutes. In addition, two transfusion ratios were calculated, including RTP: number of transfused patients x 100/number of surgeries performed and the C/T ratio.

To evaluate RBC leftovers, the number of requested bags was calculated, subtracted from the number of bags used and divided into five categories: lacking (indicating that the number of bags requested was less than the number of bags used), zero (indicating that the number of used and requested bags was equal), one (one bag was not used), two (two bags were not used) and three or more (three or more bags were not used).

In the statistical analysis, the sociodemographic data, laboratory data, data on request and use of blood products are presented as frequency and percentage; mean, standard deviation and confidence interval (CI); or median, minimum and maximum for asymmetric according to the type of variable and symmetry. The symmetry of the data was analyzed by using the *Kolmogorov-Smirnov* test, in which the data were considered asymmetrical with *p*<0.05. Chi-square, one-way ANOVA and *Kruskal-Wallis* nonparametric tests were used to compare the surgical specialties.

A binary logistic regression was performed to determine the extent that sociodemographic, laboratory and surgical characteristics influence blood product requests. The selected variables were included by the *Backward Wald* method. The *Omnibus chi-square test* (*p*<0.05) and the *Hosmer and Lemeshow test* (*p*>0.05) were used to determine the *fit* of the model, while *Nagelkerke's R^2^ test* was used with the explained index of variance, where the higher the R^2^ value is, the better the model. Variable coding and statistical analyses were conducted with *Statistical Package for the Social Sciences* (SPSS), version 23.0. The significance level adopted for all analyses was *p*<0.05.

## RESULTS

During the study period, 8,964 surgical procedures were performed, of which 1,494 procedures required a surgical reserve. Of these procedures, 822 cases were included, representing 55.02% (822/1,494) of the total procedures with blood product reserves. Table 1 presents the demographic and laboratory data results according to surgical specialties.

The mean Hb (12.6 g/dl; 95% CI: 12.3; 12.9) and Ht levels (37.28%; 95% CI: 36.9; 37.6) in this sample are similar to the reference values. However, lower values were observed, indicating mild anemia in vascular surgery, digestive tract and thoracic surgery patients. All patients of all specialties presented platelet counts within normal limits, with mean values above 150,000 platelets/mm^3^. In the coagulation screening tests (PT and APTT), altered values were observed for only 5.5% of the sample evaluated, with a predominance occurring in cardiac and digestive tract surgery cases (9.4% and 10.5%, respectively). In turn, IAS was negative in 100% of the samples evaluated (Table 1).

Table 2 shows the number of blood product units that were requested for reserve and the number of blood products used during the surgical procedure or within 24 hours of the procedure in each surgical specialty. Only 16.4% of the patients received a transfusion using RBCs, and of these patients, 67 used two units of RBC (19 received 1 IU, 37 received 03 IU, 10 received 04 IU, and only two patients received 5 IU). In cardiac surgeries, 50% of the patients RBC transfusions, with a mean of 1.15 units (one surgery received five units). In digestive tract surgeries, 25% of the patients required an RBC transfusion (mean and median: 0, minimum: 0; maximum: 4 units). RBC transfusions were utilized in 16.5%, 11%, 9.5% and 8.1% of vascular, gynecologic, urologic and thoracic surgeries, respectively, with a maximum of five RBC units used in urology surgeries, four RBC units in the vascular surgeries and three RBC units in the other specialty surgeries.

Table 3 shows the logistic regression analysis results. The independent variables (age, sex, weight, Hb, Ht, anemia, coagulopathy, duration of surgery and the six surgical specialties) influenced 27.5% of the variance in the use of RBCs (Hosmer and Lemeshow *p*=0.55; Omnibus Test of model, *p*<0.001). The age, time of surgery and cardiac surgery variables, when adjusted for all other variables in the model, significantly increased the chance of using RBCs. The older the patient (OR 1.025, 95% CI 1.006, 1.045) and the longer the surgery time was (OR 1.004, 95% CI 1.001, 1.006), the greater the chance of using RBCs. The only specialty that significantly influenced the use of RBCs was cardiac surgery, which increased the chance of using RBCs (OR 7.83 95% CI 1.58, 38.74) by 7.8-fold.

The frequencies of bags that were not used per specialty are presented in Table 4. In all, 2,175 RBC units were requested and not used for 822 procedures. Apart from gynecology, in which two units were not used, all other specialties did not use three or more RBC units. Finally, the RTP and C/T indices and the results of the procedures with a minimum frequency of 12 surgeries are demonstrated in Table 5. The RTP was greater than 10% in 22 types of surgeries, indicating the need for bag preparation. In the other 24 surgery types, a value lower than 1% was observed, not recommending any previous preparation, suggesting a waste of the preparation of packed RBCs. In addition, the C/T ratio was higher than 2.5 in 88% of the sample, which shows the excess preparation of blood products. Notably, because the frequencies of different vascular surgery types were less than 12, these surgeries were not discriminated in Table 5.

## DISCUSSION

The present study analyzed the profile of the request and use of blood products for elective surgeries in different specialties. Of the 822 procedures studied, 135 were transfused (16.4%), and in most surgeries, the requested blood products were not used, presenting a median of zero in both the total sample and each surgical specialty, even considering a postoperative period of up to 24 hours. However, among the specialties, cardiac surgeries had the highest transfusion rate, in which 50% of the patients in this specialty were transfused. This result was also reported by Stanworth et al. in 2002 14, who observed higher transfusion rates in cardiac and orthopedic procedures; however, studies have shown that the demand for and use of RBCs in cardiac surgeries may vary between medical teams and between institutions 15.

In most transfused cases (87%), at least two units were used, a result similar to that obtained in Austria by Gombotz et al. 1 in a study with 6,530 patients, which demonstrated that more than 80% of cases received the transfusion of at least two RBC units. Importantly, blood transfusions expose patients to several antigens, risk of adverse events and the possibility of acquiring infections 8,16. The current recommendation is to try to modify this paradigm and decrease the exposure of patients to blood products 17.

Another fact that was highlighted in the present study is the remaining RBC reserves. Of the 2,483 RBC units prepared, only 314 units were transfused (12.4%), leaving 87.6% unused. Similarly, Collins et al. 4 evaluated 1,350 surgical procedures and observed that 72% of RBC units prepared for these surgeries were not used. Other studies have shown use in 40-60% of patients 1,8,18.

As previously demonstrated, most prepared bags were not used, and in 66.6% of the cases, three or more RBC units remained unused. Similar data have been reported in the literature for approximately 40 years, which reinforces the need for each institution to continuously analyze preoperative RBC data on request and use 13 and to reformulate the standards for requesting blood products in hospital elective surgeries.

These data are of great relevance for hemotherapy service, as this service determines the specialties that need priority and elaborate new operational flows.

The other objective of this study was to verify the influence of the independent variables on the use of the RBCs. Age, time of surgery and cardiac specialty were the only variables that significantly influenced the amount of RBCs used. The risk of RBC transfusion increased with age in the investigated population, cardiac surgeries increased the chance of transfusion by 7.8-fold, and with each hour of surgery, the risk of transfusion increased by 24%. These results are in line with the findings of Stanworth and colleagues 14 on cardiac procedures and of Gombotz et al. 1 and Mahar et al. 3, who noted an increased need for transfusions in patients over 65 years of age.

Although there is reference to female sex as a risk factor for the use of RBCs in elective surgeries 1, in this study, gender was not a predictor for transfusion.

Notably, although preoperative anemia is described as more prevalent in patients receiving RBC transfusions 1,2,3,18, in the studied sample, the mean Hb dosage value was 12.6 g/dl, which represents values that were normal or close to normal for both sexes. Based on the Hb value range in the sample, anemia severity classifications did not influence RBC use.

Another characteristic studied was the presence of coagulogram changes (PT and APTT). These changes suggest the presence of a coagulation disorder 20. Coagulopathies expose patients to a greater risk of bleeding during the preoperative period, which positively influences the use of blood products 1,18. In this study, less than 6% of the sample evaluated presented coagulogram changes (PT and APTT); the presence of changes in the coagulograms was not identified as transfusion predictors. In another study conducted at a university hospital in Iran, Yazdi et al. 21 did not find a relationship between changes in PT and intraoperative blood use when analyzing the use of blood in surgeries for 398 patients. In this same study, Hb, Ht and platelet counts did not influence the number of units transfused during surgery. However, the literature points to several factors associated with increased perioperative transfusion risk, such as intraoperative volume loss, preoperative anemia and female sex 1,18. Nuttall et al. 22 demonstrated that the preoperative Hb level in surgical reoperation, estimated blood loss and weight are factors associated with transfusion in total hip arthroplasty surgeries. Knowledge of the data that can influence intraoperative transfusions helps clinicians make more rational decisions regarding the requests for surgical blood product reserves.

Transfusion indices may help in the management of RBC preparation for elective surgeries 6,9,10,11. Collins et al. 4 used the C/T ratio in the evaluation of excessive blood product requests for elective surgeries. Of the seven surgical specialties evaluated, only three had a C/T ratio lower than three. The authors argued that the evaluation of intraoperative blood product consumption combined with the involvement of surgical teams in the preoperative request process of blood products may reduce the excess number of RBCs required for surgical reserve.

In this study, among the 51 types of surgical procedures evaluated, 22 procedures presented an RTP >10%, indicating the need for RBC concentrates compatibility to surgical reserve. In the other 24 procedures, the RTP values were <1%, indicating that there was no need for any prior RBC preparation. In addition, we also highlighted the high C/T values found in transfusion surgeries, where 88% of the procedures presented a C/T ratio greater than 2.5, corroborating our hypothesis that many RBC units are unnecessarily prepared. We believe that if several subjects were not excluded, we would have a stronger sample size that would yield even more significant results. These data demonstrate the need for new institutional protocols.

## CONCLUSION

Most surgeries did not use the requested blood products. Age, time of surgery and cardiac surgeries increased the chance of RBC use. RTP and C/T may guide the request for RBCs during the preoperative period of elective surgeries.

The blood reserve request patterns for elective surgeries contribute to stock management and operational resource optimization. The verification of blood consumption between different medical specialties provides data that can direct the application of good practice strategies in hemotherapy. The development of protocols for blood reserve for elective surgeries becomes necessary in institutions.

## AUTHOR CONTRIBUTIONS

All of the authors participated meaningfully in the study and that they have read and approved the final version of the manuscript. The coauthors Carrareto AR, Zago-Gomes MP, Orletti MP and Novaes AC participated in the data collection and preparation of work.

## Figures and Tables

**Table 1:** Demographic and laboratory characteristics of patients in the surgical specialties evaluated.

Variables		Total Sample (%)	Specialties						*p*
			Cardiac	Urology	Gynecology	Digestive Syst.	Vascular	Thoracic	
Participants		822 (100)	106 (12.9)	308 (37.5)	181 (22)	157 (19.1)	30 (3.6)	37 (4.5)	
Sex									
M		410 (49.9)	63 (59.4)	230 (74.7)	0	75 (47.8)	14 (46.7)	26 (70.3)	0.00**
F		412 (50.1)	43 (40.6)	78 (25.3)	181 (100)	82 (52.2)	16 (53.3)	11 (29.7)	
Coagulogram changes (PT and APTT)									
Yes		38 (5.5)	9 (9.4)	9 (3.7)	4 (2.6)	22 (10.5)	1 (4.3)	1 (3.1)	0.03**
No		648 (94.5)	87 (90.6)	237 (96.3)	151 (97.4)	119 (89.5)	22 (95.7)	31 (96.9)	
	N		Mean±SD (IC)						
Age (years)	822	55.4±15.45	56.62±11.74	59.56±15.10	46.70±12.34	54.55±15.29	64.93±13.98	48.04±19.73	0.00***
		(54.08-56.20)	(54.28-58.96)	(57.23-61.90)	(44.07-49.33)	(51.40-57.70)	(57.19-72.67)	(39.29-56.79)	
Hemoglobin (g/dl)	809	12.6±4.4	12.69±1.95	12.90±1.5	12.25±1.7	11.85±2.03	11±2.2	11.91±2.11	0.00***
		(12.32-12.93)	(12.31-13.07)	(12.72-13.08)	(11.99-12.51)	(11.34-13.38)	(10.28-11.93)	(11.20-12.61)	
Hematocrit (%)	809	37.28±5.36	37.95±5.87	38.82±4.56	36.90±4.94	35.23±5.61	33.62±6.1	36.26±5.7	0.00***
		(36.91-37.65)	(36.81-39.08)	(38.30-39.34)	(36.17-37.63)	(34.35-36.12)	(31.34-35.90)	(34.34-38.18)	
Platelet count (mil/mm^3^)	790	255.000±98.960	231.540±0.030	225.300±77.900	269.410±94.270	263.890±115.980	301.26±140.880	331.210±150.960	0.00***
		(242.007-255.919)	(216.054-247.030)	(216.437-234.171)	(255.000-283.355)	(245.607-282.176)	(248.660-53.872)	(280.882-381.549)	

Syst.: System; PT: prothrombin time; APTT: activated partial thromboplastin time; **χ^2^; ***ANOVA.

**Table 2:** Results of the median request and use of blood products (in units) in the surgical specialties evaluated.

Variables	N	Surgical SpecialtiesMedian (minimum-maximum)							*p*
		Total Sample	Cardiac	Urological	Gynecological	Digestive system	Vascular	Thoracic	
RBC requested	822	3 (0-8)	4.0 (2-8)	3.0 (2-5)	2.0 (1-4)	3.0 (2-3)	3 (0-5)	3 (2-5)	0.00****
RBC used	822	0 (0-5)	0 (0-5)	0 (0-5)	0 (0-3)	0 (0-4)	0 (0-4)	0 (0-3)	0.00****
RBC used in 24 hours	818	0 (0-3)	0 (0-3)	0 (0-2)	-	0 (0-3)	0 (0-2)	-	0.00****
PC requested	822	0 (0-10)	8 (0-10)	0 (0 -7)	0 (0-3)	0 (0-8)	0 (0-8)	0 (0-8)	0.00****
PC used	822	0 (0-10)	0 (0-8)	-	-	0 (0-10)	0 (0-7)	-	0.00****
FFP requested	822	3 (0-10)	8 (0-10)	3 (0-10)	0 (0-3.0)	3.0 (0-3.0)	3 (0-5)	0 (0-4)	0.00****
FFP used	822	0 (0-10)	0 (0-10)	0 (0-10)	0 (0-3)	0 (0-3)	0 (0-4)	0 (0-3)	0.15****

PC: Platelet concentrate; FFP: Fresh frozen plasma; ****Kruskal-Wallis.

**Table 3:** Binary logistic regression to detect the influence of demographic, clinical and laboratory variables on the use of RBCs in the surgical procedures studied.

Independent variables	OR	CI 95%	*p*
Sex	1.619	0.89-2.92	0.11
Weight	0.991	0.97-1.01	0.34
Age	1.025	1.00-1.04	0.01*
Mild anemia			0.51
Moderate anemia	0.837	0.31-2.25	0.72
Severe anemia	3.694	0.22-60.35	0.35
Hemoglobin	0.910	0.65-1.26	0.57
Hematocrit	0.920	0.82-1.034	0.16
Coagulopathy	1.590	0.58-4.35	0.36
Surgery time	1.004	1.001-1.006	0.00*
Cardiac surgery	7.830	1.58-38.74	0.01*
Urology	1.246	0.25-6.11	0.78
Surgery Digestive system	0.756	0.14-4.01	0.74
Gynecology	1.850	0.35-9.73	0.46
Vascular	0.793	0.10-6.01	0.82

OR - Odds Ratio; CI - Confidence interval.

*p<0.05.

**Table 4:** Frequency of leftover RBC units in surgical procedures by specialty.

Specialties	N	Lacking N (%)	Zero N (%)	One N (%)	Two N (%)	3 or more N (%)
Urological Surgery	308	2 (0.6)	19 (6.2)	3 (1)	16 (5.2)	268 (87)
Gynecological Surgery	181	1 (0.6)	17 (9.4)	6 (3.3)	108 (59.7)	49 (27.1)
Surgery Digestive system	157	2 (1.3)	10 (6.4)	6 (3.8)	6 (12.1)	120 (76.4)
Cardiac surgery	106	0	8 (7.5)	2 (1.9)	25 (23.6)	71 (67)
Thoracic surgery	37	0	2 (5.4)	0	17 (45.9)	18 (48.6)
Vascular surgery	30	0	3 (10)	1 (3.3)	7 (23.3)	19 (63.3)
Total	822	5 (0.6)	59 (7.2)	18 (2.2)	193 (23.5)	547 (66.6)

**Table 5:** Analysis of transfusion data and transfusion rates in the surgical specialties studied according to surgery type.

Surgical specialties and type	N	No. of patients transfused	No. of transfused units	RTP	C/T
**Urological surgeries**					
Radical Prostatectomy	92	7	14	7.61	19.5
Transvesical Prostatectomy	67	8	23	11.94	8.74
Nephrectomy	67	10	26	14.93	7.65
Percutaneous Nephrolithotripsy	45	0	0	-	-
Urinary tract procedure	20	1	2	5.00	29
**Gynecological Surgeries**					
Total Abdominal Hysterectomy	92	10	20	10.87	10.55
Gynecological Exploratory Laparotomy	41	3	6	7.32	15.6
Vaginal Hysterectomy	18	2	4	11.11	9
Uterine Curettage	16	4	9	25.00	3.78
**Digestive system surgery**					
Colectomy	48	8	17	16.67	7.94
General Exploratory Laparotomy	31	2	3	6.45	83
Gastrectomy (includes esophagectomy)	20	4	9	20.00	6.56
Whipple Surgery	14	3	7	21.43	6
Biliodigestive Derivation	11	2	6	18.18	5.33
**Cardiac surgeries**					
Myocardial revascularization	69	37	87	53.62	3.95
Valvular Surgery	31	15	35	48.39	4.14
**Thoracic surgery**					
Mediastinoscopy (includes pleuroscopy, pulmonary decortication and pleurodesis)	19	1	2	5.26	26

RTP: rate of transfused patients; C/T: RBC cross-matched/transfused RBC.
